# Exploring the application value of pro‐gastrin‐releasing peptide in the clinical diagnosis and surgical treatment of medullary thyroid carcinoma

**DOI:** 10.1002/cam4.6572

**Published:** 2023-09-27

**Authors:** Qiang Miao, Xiaohui Lv, Li Luo, Junlong Zhang, Bei Cai

**Affiliations:** ^1^ Department of Laboratory Medicine/Research Center of Clinical Laboratory Medicine West China Hospital of Sichuan University Chengdu China

**Keywords:** calcitonin, carcinoembryonic antigen, efficacy assessment, medullary thyroid carcinoma, pro‐gastrin‐releasing peptide, tumor metastasis

## Abstract

**Objective:**

To investigate the relationship between pro‐gastrin‐releasing peptide (ProGRP) and the clinical characteristics of patients with medullary thyroid carcinoma (MTC) and the value of ProGRP in surgical treatment monitoring.

**Patients and Methods:**

A total of 347 patients with MTC and non‐MTC malignant and benign thyroid diseases were enrolled. The concentrations of neuron‐specific enolase (NSE), carcinoembryonic antigen (CEA), calcitonin (CT), and ProGRP were determined by Elecsys® assays. The NSE, CEA, CT, and ProGRP levels in different thyroid disease groups were compared, and ProGRP levels in different clinicopathological feature groups pre and postoperatively were further compared.

**Results:**

The CT, CEA, NSE, and ProGRP levels were upregulated in the MTC group compared to those in the non‐MTC malignant and benign thyroid disease groups. The area under the receiver operating characteristic curve (AUC) of ProGRP for the diagnosis of MTC was 0.832(0.787–0.871), similar to that of CT and CEA. The sensitivity and specificity were 71.4% and 92.7%, respectively, and the optimal cut‐off value was 61.8 pg/mL. The AUC of ProGRP combined with CT or CEA for the diagnosis of MTC was 0.933 (0.900–0.958) and 0.922 (0.886–0.949), respectively, which were higher than those of a single index. ProGRP levels were higher in patients with lymph nodes and distant metastases than in patients without metastases. The postoperative level of ProGRP was lower than that before treatment.

**Conclusion:**

ProGRP is comparable to CEA and CT as an MTC biomarker with broad prospects. It has potential application value in the progression of MTC assessment and the evaluation of surgical intervention effects.

## 1 INTRODUCTION

Thyroid cancer is the most common endocrine tumor and is more prevalent in women than in men. Recently, with the increasing popularity and sensitivity of ultrasound examinations and the increase in other diagnostic imaging methods, its detection rate has rapidly increased.^1^ Thyroid carcinoma can be classified into papillary thyroid cancer, follicular thyroid cancer, poorly differentiated thyroid cancer, anaplastic thyroid cancer, and medullary thyroid carcinoma (MTC), according to its pathological type.^2^ Medullary thyroid carcinoma is a rare malignant tumor of the neuroendocrine system originating, from calcitonin‐secreting cells (C or parafollicular cells) in the thyroid. MTC accounts for 5%–10% of malignant thyroid tumors and 13.4% of fatal thyroid cancer. Based on its genetic characteristics, it can be classified into sporadic and hereditary MTC, with incidence rates of approximately 75% and 25%, respectively.^3^, ^4^, ^5^ Furthermore, it is prone to lymph nodes invasion and distant metastasis at an early stage; therefore, its prognosis is relatively poor. The ideal survival period of patients with MTC mainly depends on the time from diagnosis to the application of treatment strategies.^6^, ^7^, ^8^ Therefore, early diagnosis and management of MTC are crucial.

An accurate preoperative diagnosis of MTC can assist surgeons in optimizing the surgical plan and evaluating the prognosis. Currently, the diagnosis of MTC mainly relies on medical history and physical examination, ultrasound and ultrasound‐guided fine‐needle aspiration pathological biopsy (FNAB), screening for ret gene mutations, and the serological marker calcitonin (CT).^9^, ^10^ However, these methods have limitations. For example, CT may increase in chronic thyroiditis and C‐cell hyperplasia. In addition, some studies have reported the presence of serum CT‐negative non‐secretory MTC, accounting for 12% of all MTC cases.^11^ The standard method for diagnosing MTC is currently the cytological evaluation of FNAB thyroid lesions; up to 30% of FNAB results are uncertain, leading to the need for repeated FNAB or tumor surgery to determine the diagnosis.^2^, ^12^ Some studies have shown that pro‐gastrin‐releasing peptide (ProGRP) may also be helpful in the diagnosis of MTC and in monitoring treatment response.^13^, ^14^, ^15^ However, because of the low incidence of MTC, only a limited number of assessments have been conducted. Therefore, the purpose of this study was to evaluate the ability of ProGRP and other indicators, alone or in combination, to diagnose MTC and to explore the relationship between ProGRP and the clinical characteristics of patients with MTC as well as the value of ProGRP in evaluating the effect of surgical treatment.

## 2 METHODS

### 2.1 Study population

Patients with thyroid nodules who visited our hospital from June 2020 to December 2021 and underwent ultrasound‐guided thyroid nodule FNAB were included. Clinical data and laboratory examination results were recorded. Patients with other malignant tumors or abnormal liver and kidney functions were excluded. Moreover, a total of 347 patients were included in the study. The participants were classified into three groups: MTC, non‐MTC malignant, and benign thyroid disease. The MTC group included 100 patients with MTC. The benign thyroid disease group included patients with benign thyroid nodules, hyperthyroidism, hypothyroidism, or goiter (*n* = 111). Moreover, the non‐MTC malignant thyroid disease group included patients with papillary thyroid carcinomas and follicular carcinomas (*n* = 136). Among them, there were 117 cases without lymph node metastasis and 19 cases with lymph node metastasis. The basic demographic information of the study participants is presented in Table 1. Simultaneously, the residual serum samples from the study participants who underwent tumor marker detection in our laboratory were used to determine ProGRP and neuron‐specific enolase (NSE) concentrations. All participants signed informed consent before residual serum samples were collected. The study protocol was approved by the West China Hospital Ethics Committee of Sichuan University (No.2020‐823). All methods were performed according to the relevant guidelines and regulations.

**TABLE 1:** Comparison of CEA, NSE, ProGRP, and CT in peripheral blood between different groups.

Parameters	Benign thyroid disease (*n* = 111)	Non‐MTC malignant tumor (*n* = 136)	MTC (*n* = 100)	*p*	Adjust p*
Age (years)	46.0 (34.0, 54.0)	39.0 (32.0, 49.0)^c^	48.5 (39.0, 57.7)^b^	0.000	–
Sex (male/female)	28/83	40/96	40/60	0.059	–
CT (pg/mL)	1.06 (0.5, 2.65)	0.86 (0.5, 1.92)	383.55 (48.68, 4319.25)^a^, ^b^	0.000	0.000
CEA (ng/mL)	1.36 (0.89, 1.94)	1.37 (0.9, 1.95)	11.70 (3.55, 84.5)^a^, ^b^	0.000	0.000
NSE(ng/mL)	12.5 (11.3, 14.95)	11.7 (9.99, 13.7)	14.65 (12.4, 17.3)	0.000	0.234
ProGRP (pg/mL)	41.85 (34.63, 52.28)	35.6 (28.70, 42.9)	103 (52.27, 1099.75)^a^, ^b^	0.000	0.000

*Note*: Data are summarized as median (interquartile range) for continuous variables and number for categorical variables. Kruskal–Wallis test was used for multiple group comparison analysis and Dunn–Bonferroni test was used for post hoc comparisons. Adjust p*: Results of the Kruskal–Wallis test after using the analysis of covariance (ANCOVA) to control the effects of age‐confounding variables. Results of pairwise comparisons.

Abbreviations: CEA, carcinoembryonic antigen; CT, calcitonin; MTC, medullary thyroid carcinoma; NSE, neuron‐specific enolase; ProGRP, pro‐gastrin‐releasing peptide.

^a^ *p* < 0.05 versus Benign thyroid disease group post hoc comparisons.

^b^ *p* < 0.05 versus non‐MTC malignant tumor group post hoc comparisons.

^c^ *p* < 0.05 versus benign thyroid disease group post hoc comparisons.

### 2.2 Study design

The grouping of participants was based on a longitudinal review of clinical, imaging, biochemical (including serum CT and carcinoembryonic antigen [CEA] levels), and available cytology/histological data The tumor‐node‐metastasis (TNM) staging of cancer in patients followed the current American Joint Committee on Cancer staging system (8th edition) for MTC.^16^ T1 indicates that the tumor is confined to the thyroid gland, its tumor size being <2 cm, and >T1 stands for a tumor size ≥2 cm, a tumor infiltrating outwards into the thyroid, lymph node metastasis, or distant metastasis. We further classified patients with MTC into subgroups based on their clinical and pathological characteristics. These pathologies include in situ tumors, lymph node metastases, distant metastases, clear nodule boundary, and unilateral or bilateral lesions. In addition, the levels of ProGRP in 100 patients with MTC before and 1 week after surgery were compared.

### 2.3 Determination of relevant index concentration

The plasma CT concentration was measured by a fully automated electrochemiluminescence immunoassay (ECLIA) Cobas® e601 (Roche Diagnostics GmbH). Furthermore, serum CEA, NSE, and ProGRP concentrations were measured using an ECLIA Cobas® e801 (Roche Diagnostics). Moreover, all reagents, calibrators, and quality control materials were purchased from Roche Diagnostics (Basel, Switzerland). In this study, results that were less than the lower limit of instrument detection were recorded as the lowest detection limit. In contrast, results higher than the upper limit of instrument detection were quantitated by dilution according to the dilution ratio recommended in the reagent manual.

### 2.4 Statistics analysis

Statistical analyses were performed using Social Science Program Statistical Software Package (IBM SPSS Statistics for Windows, Version 22.0. Armonk, NY: IBM Crop) and GraphPad Prism version 7.00 (GraphPad Software). The analysis of area under the receiver operating characteristic (ROC) curves (AUC) was performed with the MedCalc® statistical software version 19.4.1 (MedCalc® Software Ltd., Ostend, Belgium; 2020). Values are presented as the mean ± standard deviation for normally distributed data, median, and interquartile range for data that were non‐normally distributed for continuous variables, and numbers for categorical variables. Additionally, analysis of covariance (ANCOVA) was used to control the effects of age‐confounding variables. Furthermore, the Mann–Whitney *U* and Kruskal–Wallis tests were used to compare the means of two and multi‐group variables that were not normally distributed. The Dunn–Bonferroni test was used for post hoc comparisons. For all tests, a *p* < 0.05 was considered statistically significant.

## 3 RESULTS

### 3.1 Demographic data and analysis of serum tumor marker levels between groups

After application of inclusion/exclusion criteria, 347 patients were included in our study cohort. There were significant differences in age among the three groups (*p* = 0.000). Pairwise comparisons showed that the MTC group was older than the non‐MTC group (*p* < 0.05); however, there was no significant difference between the MTC and benign disease groups. Notably, women accounted for 66.1% (156/236) of the patients with thyroid cancer. There was no statistically significant difference in sex among the three groups (*p* = 0.059). After adjusting for age as a confounding factor, there was no difference in NSE levels among the three groups (*p* = 0.234); in contrast, there were still differences in CEA, CT, and ProGRP levels among the three groups (*p* = 0.000, 0.000, and 0.000, respectively). In addition, pairwise comparisons showed that the CEA, CT, and ProGRP levels were higher in the MTC group than in the non‐MTC and benign disease groups. Further details are presented in Table 1.

### 3.2 The diagnostic performance of CEA, NSE, ProGRP, and CT in MTC diagnosis

**FIGURE 1:**
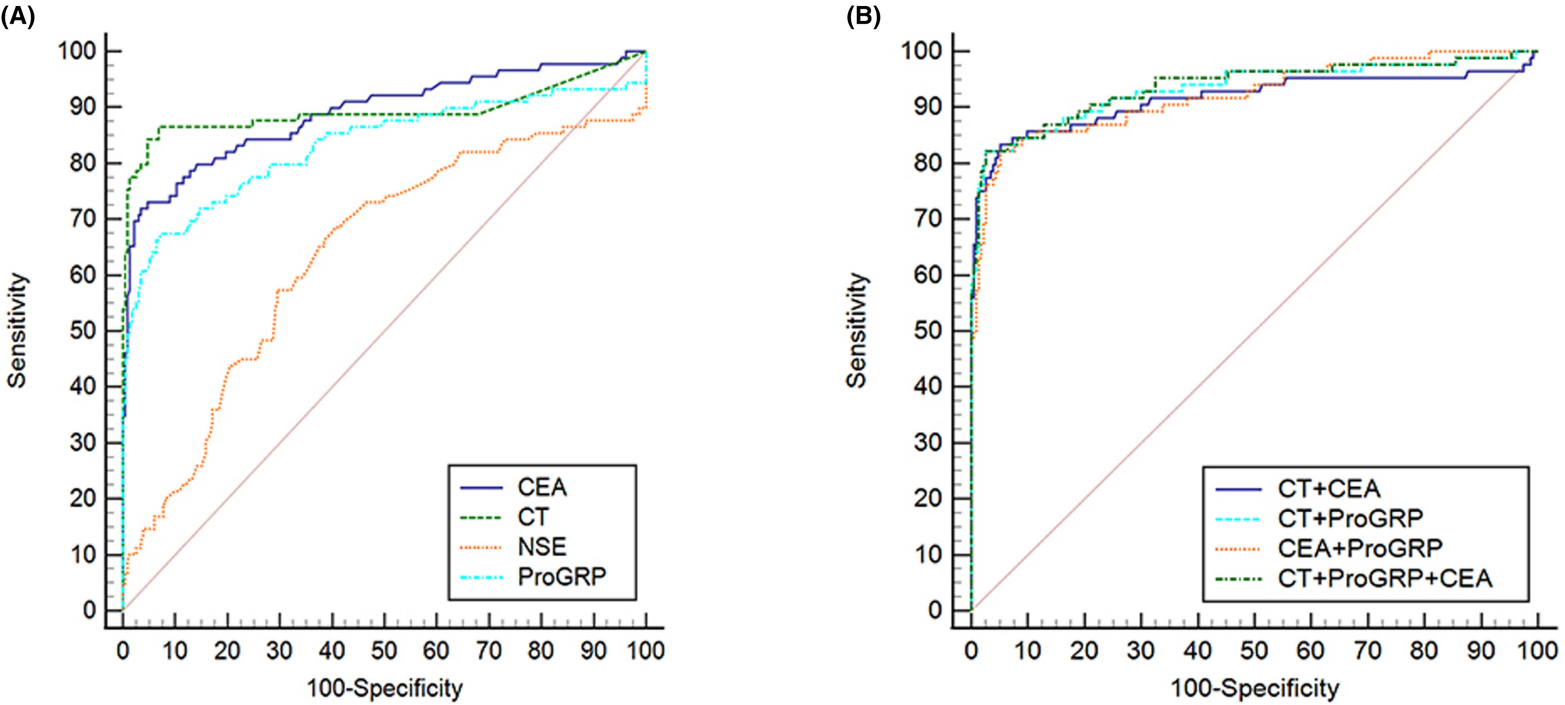
Receiver operating characteristic curve analysis of serum tumor markers in the diagnosis of MTC. (A) Comparison of the value of single index in the diagnosis of MTC; (B) Using joint analysis to compare the value of Pro‐GRP in diagnosing MTC. CT, calcitonin; CEA, carcinoembryonic antigen; NSE, neuron‐specific enolase; ProGRP, pro‐gastrin‐releasing peptide.

The ROC curve analysis showed that the AUC of ProGRP for the diagnosis of MTC was 0.832, similar to that of CT (0.893) and CEA (0.890). This indicated that ProGRP had a diagnostic value for MTC. The sensitivity and specificity of ProGRP were 71.4% and 92.7%, respectively, and the optimal cut‐off value was 61.8 pg/mL. The AUC of NSE for MTC diagnosis was 0.638, indicating poor diagnostic ability. Furthermore, the AUC of ProGRP combined with CT or CEA in the diagnosis of MTC was 0.933 and 0.922, respectively, and the AUC of CT combined with CEA in the diagnosis of MTC was 0.915, which was higher than that of the single indices (all *p* < 0.05). Additionally, the AUC of the CT, CEA, and ProGRP combination for MTC diagnosis was 0.937, similar to that of the bi‐index combination (Table 2; Figure 1).

**TABLE 2:** Sensitivity and specificity of all tumor markers in MTC, individually and in combination.

	Cut‐off value	Sensitivity (%)	Specificity (%)	AUC (95% CI)
CEA	4.73 ng/mL	72.6	97.9	0.890(0.850–0.922)
CT	7.97 pg/mL	85.7	93.2	0.893(0.854–0.925)
NSE	12.75 ng/mL	71.4	59.4	0.638(0.583–0.690)
ProGRP	61.80 pg/mL	71.4	92.7	0.832(0.787–0.871)
CT + CEA		83.3	94.9	0.915(0.879–0.944)
CEA + ProGRP		82.1	94.4	0.922(0.886–0.949)
CT + ProGRP		82.1	97.4	0.933(0.900–0.958)
CT + ProGRP + CEA		82.1	97.4	0.937(0.904–0.961)

Abbreviations: CEA, carcinoembryonic antigen; CI, confidence interval; CT, calcitonin; MTC, medullary thyroid carcinoma; NSE, neuron‐specific enolase; ProGRP, pro‐gastrin‐releasing peptide.

### 3.3 Serum ProGRP could assess the severity of MTC

**FIGURE 2:**
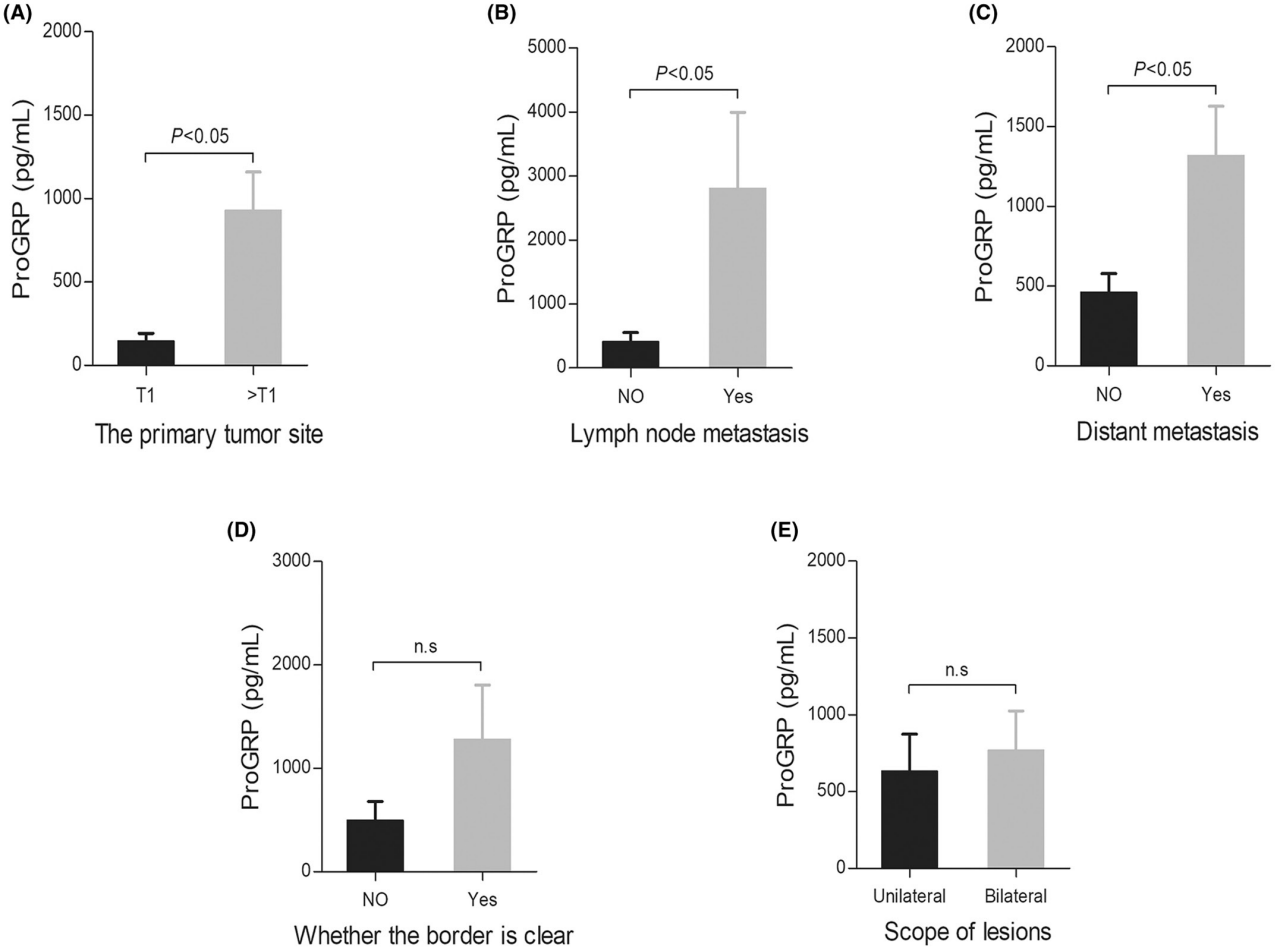
Comparison of ProGRP median levels at different severities of MTC by Mann–Whitney *U*‐test. (A) Compared the ProGRP concentration in the primary tumor site: T1 group (41 cases) versus >T1 group (59 cases); (B) Compared the ProGRP concentrations in lymph node metastases: without lymph node metastases group (34 cases) versus with lymph node metastasis group (66 cases); (C) Compared the ProGRP concentrations in distant metastases: without distant metastases group (70 cases) versus with distant metastases group (20 cases); (D) Compared the ProGRP concentrations in whether the boundary is clear: obscure boundary group (76 cases) versus clear boundary group (24 cases); (E) Compared the concentrations of ProGRP in unilateral and bilateral lesions: unilateral lesions group (60 cases) versus bilateral lesions group (40 cases). n.s: *p* > 0.05. MTC, medullary thyroid carcinoma; ProGRP, pro‐gastrin‐releasing peptide.

We further investigated ProGRP levels at different severities of MTC (Figure 2). The results showed that the level of ProGRP in patients with tumor stage >T1 was significantly higher than that in patients with T1 stage (*p* < 0.05; Figure 2A). Similarly, ProGRP levels were significantly higher in patients with lymph node metastasis and distant metastasis than in those without metastasis (*p* < 0.05; Figure 2B,C, respectively). Furthermore, there was no significant difference in ProGRP levels between the clear and unclear boundary groups or between the unilateral and bilateral groups regarding the scope of the lesions (*p* > 0.05; Figure 2E,F, respectively).

### 3.4 Serum ProGRP could evaluate the effects of surgical treatment of MTC

**FIGURE 3:**
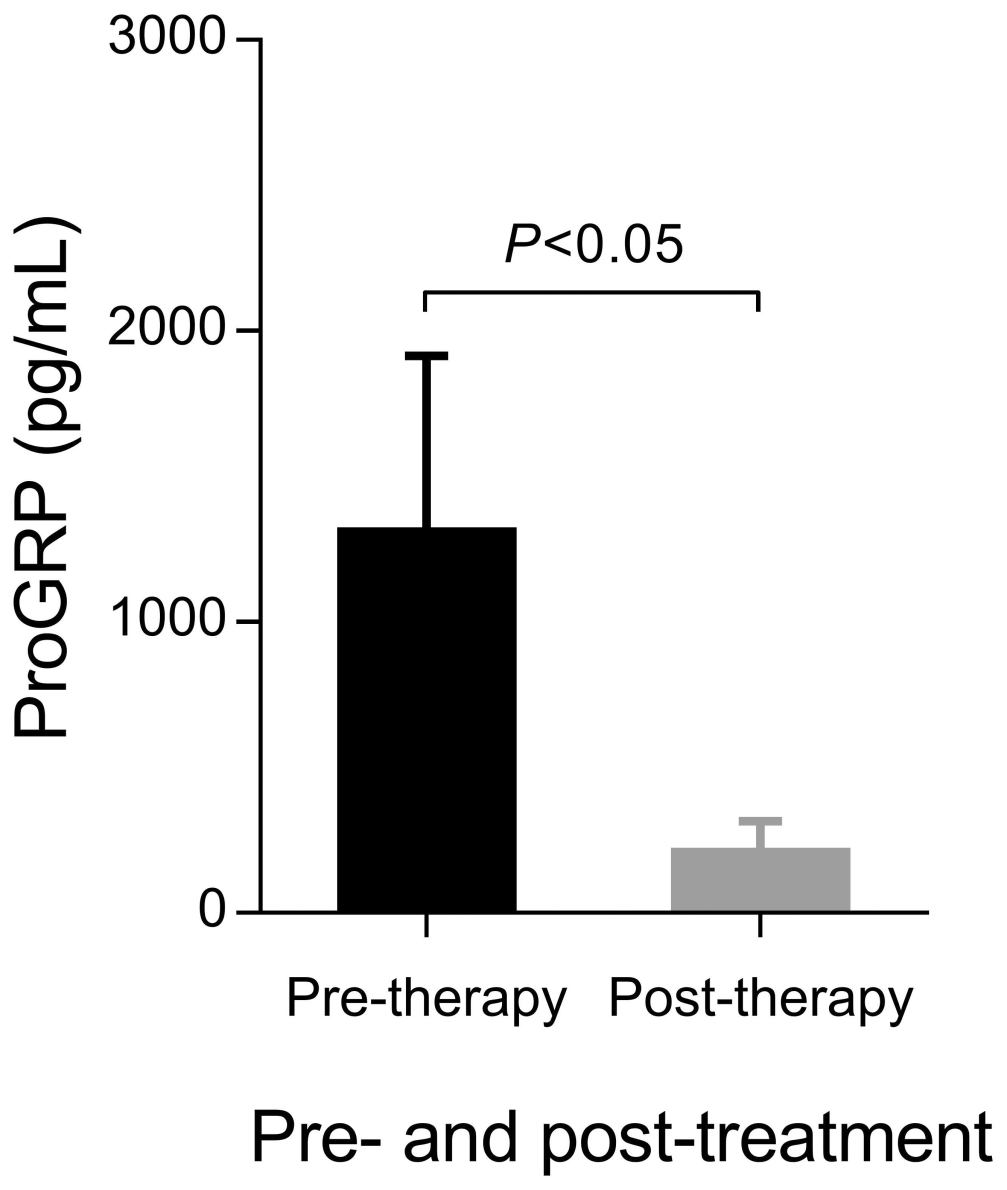
Comparison of median levels of ProGRP before and 7 days after surgery in patients with MTC. MTC, medullary thyroid carcinoma; ProGRP, pro‐gastrin‐releasing peptide.

To further investigate the potential of ProGRP as a surgical intervention assessment index, we analyzed ProGRP levels in patients before and 1 week after surgery. The results showed that the level of postoperative ProGRP was significantly decreased compared with the preoperative level (*p* < 0.05, Figure 3). This showed that ProGRP helps evaluate the therapeutic effect of MTC surgery and can be used as a potential marker for postoperative monitoring.

## 4 DISCUSSION

The incidence of thyroid cancer is related to sex and usually occurs in females.^1^ Notably, similar observations were made in the present study. The proportion of females with thyroid cancer (including MTC and non‐MTC tumors) was higher than that of males. MTC is highly malignant and prone to lymph node and distant metastases at an early stage.^7^ About 1/3 of MTC patients will relapse, and patients with a high tumor load at diagnosis are more likely to relapse or survive with a tumor. Recently, ProGRP has been used as a biomarker for small‐cell lung cancer and MTC.^13^, ^17^ This study revealed that the serum ProGRP level in MTC patients was higher than that in patients with non‐medullary thyroid disease, which performs well in the differential diagnosis of MTC. Moreover, ProGRP concentrations were significantly higher in patients with MTC at a higher TNM stage than in those at a lower TNM stage. In addition, the concentration of ProGRP in patients with MTC decreased significantly 1 week after surgery compared to that before surgery. In summary, serum ProGRP is a suitable tumor marker for MTC and can be used as an indicator of disease severity and surgical intervention.

As early as 2000, Inaji et al.^18^ observed that all MTC tumor tissues highly expressed ProGRP, and its concentration in the serum varied simultaneously with CEA and CT. Furthermore, Parra‐Robert et al.^15^ observed that the median concentrations of serum CT, CEA, and ProGRP were significantly higher in patients with advanced MTC. In this study, we also observed that the levels of serum CT, CEA, and ProGRP in the MTC group were much higher than those in the non‐MTC malignant tumor and benign thyroid disease groups. The AUCs of single CT, CEA, and ProGRP were similar, all of which were >0.8, with good diagnostic efficacy for MTC, consistent with previous reports.^13^, ^18^ However, the diagnostic performance of serum NSE levels in MTC remains poor. This is consistent with the findings of Grauer et al.,^19^ who discovered that although NSE is useful for immunocytochemistry in patients with MTC, it is not a reliable serum tumor marker. In the combined diagnostic analysis, the AUCs of ProGRP combined with CT or CEA and the combination of the three indicators significantly improved, exceeding 0.9. ProGRP was proven to be an effective complementary tumor marker for MTC diagnosis, and when combined with CT or CEA, it can more accurately differentiate MTC from other thyroid cancers in the early stages.^20^

However, it is unclear whether ProGRP levels are related to MTC severity and progression. For instance, Luca Giovanella et al.^17^ reported that the concentration of serum ProGRP was significantly higher in patients with MTC and evidence of structural disease than in those without. In this study, we observed that the level of ProGRP in patients with tumor stages greater than T1 was significantly higher than that in patients with stage T1 tumors. Similarly, ProGRP levels in patients with lymph node and distant metastases were significantly higher than those without metastases. These findings suggested that preoperative ProGRP level is a potential evaluation index for TNM staging of cancer in patients with MTC and is expected to become a marker for predicting lymph nodes and distant metastases in patients with MTC. Nevertheless, additional large‐sample studies are needed to confirm the cut‐off value for the prediction of TNM staging.

It has been proposed that serum ProGRP can detect drug resistance early and evaluate its effectiveness in monitoring treatment with tyrosine kinase inhibitors in patients with advanced, inoperable MTC.^17^ In this study, we observed that the ProGRP level in patients with MTC 1 week after the operation was significantly lower than before. This shows that ProGRP is also useful in evaluating the effects of surgical intervention. However, the previous studies and ours are limited because MTC is a rare tumor with a low incidence rate; therefore, it is impossible to include sufficient samples. Another limitation of this study is that there was no follow‐up to evaluate the prognosis. Therefore, additional multicenter, large‐sample, high‐quality clinical studies are necessary to further confirm the role of ProGRP in treating and monitoring patients with MTC.

## 5 CONCLUSIONS

Serum ProGRP is comparable to CEA and CT as an MTC biomarker, with broad prospects. It has potential application value in the progression of MTC assessment and the evaluation of surgical intervention effects.

## AUTHOR CONTRIBUTIONS

**Qiang Miao:** Data curation (equal); methodology (equal); writing – original draft (equal). **Xiaohui Lv:** Software (equal); validation (equal). **Li Luo:** Data curation (equal); formal analysis (equal); investigation (equal). **Junlong Zhang:** Conceptualization (equal); project administration (equal); writing – review and editing (equal). **Bei Cai:** Project administration (equal); supervision (equal); writing – review and editing (equal).

## CONFLICT OF INTEREST STATEMENT

The authors declare no conflicts of interest.

## ETHICS STATEMENT

The study protocol was approved by the Ethics Committee of the West China Hospital, Sichuan University (No. 2020‐823). All participants provided informed consent for the use of anonymized data in this study.

## Data Availability

Data sharing does not apply to this article, as no new data were created or analyzed in this study.
